# Socioeconomic barriers to psychotherapy engagement in treatment-resistant depression

**DOI:** 10.1192/bjb.2026.10262

**Published:** 2026-08

**Authors:** Verônica de Oliveira Reis, Antonio E Nardi, Stephanie Zakhour

**Affiliations:** 1 Institute of Psychiatry, Treatment-Resistant Depression Out-patient Clinic, Federal University of Rio de Janeiro, Brazil.; 2 Laboratory of Panic & Respiration, Treatment-Resistant Depression Out-patient Clinic, Federal University of Rio de Janeiro, Brazil; 3 Treatment-Resistant Depression Out-patient Clinic, Federal University of Rio de Janeiro, Brazil

Treatment-resistant depression (TRD) is commonly defined by inadequate response to antidepressant trials, yet it remains a heterogeneous construct with ongoing debate regarding its mechanisms and boundaries.^1,2^ Although pharmacological non-response is central to current definitions, engagement with and continuity of care are also shaped by social and structural conditions, particularly in low- and middle-income countries.^3^

We report a clinical observation from a pilot implementation of a group-based psychotherapy programme delivered within a Brazilian public university hospital. The intervention was offered to six out-patients with TRD receiving care through the public health system. Participants presented marked socioeconomic vulnerability: most were unemployed, with very low monthly income, and relied on public transportation to attend sessions.

Although the intervention itself was feasible to implement within the service, sustained patient engagement proved difficult. Attendance was inconsistent, and several participants discontinued treatment. These interruptions were not primarily associated with clinical worsening but rather with practical barriers, particularly transportation costs and financial instability, which limited participants’ ability to attend regular sessions. These observations were based on a small clinical sample and are not sufficient to redefine TRD, but they highlight a clinically relevant dimension that may be under-recognised.

These findings suggest that in resource-constrained settings, apparent treatment resistance may in some cases be compounded by systemic barriers to sustained care. In such contexts, resistance to treatment may be difficult to disentangle from ongoing socioeconomic adversity that disrupts treatment continuity and exposure.^4,5^ Whereas biological and psychological mechanisms remain central to TRD, structural constraints may meaningfully influence their expression in real-world clinical settings.

From a clinical and service delivery perspective, these findings highlight the importance of adapting interventions to the realities of vulnerable populations. Strategies such as hybrid or remote delivery models, transport support and flexible scheduling may improve engagement. In addition, research in TRD may benefit from distinguishing between lack of therapeutic response and limitations in treatment continuity due to external constraints.

In low- and middle-income country settings, incorporating social determinants into both clinical formulation and service design may be essential to avoid misinterpreting disrupted treatment continuity as intrinsic treatment resistance, particularly in public sector services managing complex depressive disorders.
